# A global survey of prokaryotic genomes reveals the eco-evolutionary pressures driving horizontal gene transfer

**DOI:** 10.1038/s41559-024-02357-0

**Published:** 2024-03-05

**Authors:** Marija Dmitrijeva, Janko Tackmann, João Frederico Matias Rodrigues, Jaime Huerta-Cepas, Luis Pedro Coelho, Christian von Mering

**Affiliations:** 1 Department of Molecular Life Sciences and Swiss Institute of Bioinformatics, University of Zürich, Zurich, Switzerland; 2 Department of Biology, Institute of Microbiology and Swiss Institute of Bioinformatics, ETH Zürich, Zurich, Switzerland; 3 Centro de Biotecnología y Genómica de Plantas, Universidad Politécnica de Madrid (UPM)–Instituto Nacional de Investigación y Tecnología Agraria y Alimentaria (INIA-CSIC), Campus de Montegancedo-UPM, Madrid, Spain; 4 Institute of Science and Technology for Brain-Inspired Intelligence, Fudan University, Shanghai, China; 5 Centre for Microbiome Research, School of Biomedical Sciences, Queensland University of Technology, Translational Research Institute, Woolloongabba, Queensland Australia

**Keywords:** Molecular evolution, Computational biology and bioinformatics, Microbial ecology, Prokaryote

## Abstract

Horizontal gene transfer, the exchange of genetic material through means other than reproduction, is a fundamental force in prokaryotic genome evolution. Genomic persistence of horizontally transferred genes has been shown to be influenced by both ecological and evolutionary factors. However, there is limited availability of ecological information about species other than the habitats from which they were isolated, which has prevented a deeper exploration of ecological contributions to horizontal gene transfer. Here we focus on transfers detected through comparison of individual gene trees to the species tree, assessing the distribution of gene-exchanging prokaryotes across over a million environmental sequencing samples. By analysing detected horizontal gene transfer events, we show distinct functional profiles for recent versus old events. Although most genes transferred are part of the accessory genome, genes transferred earlier in evolution tend to be more ubiquitous within present-day species. We find that co-occurring, interacting and high-abundance species tend to exchange more genes. Finally, we show that host-associated specialist species are most likely to exchange genes with other host-associated specialist species, whereas species found across different habitats have similar gene exchange rates irrespective of their preferred habitat. Our study covers an unprecedented scale of integrated horizontal gene transfer and environmental information, highlighting broad eco-evolutionary trends.

## Abstract (web summary)

The authors analyse 8,790 prokaryotic pangenomes to identify the ecological variables associated with recent versus old horizontal gene transfer events, finding that gene transfers are more common among co-occurring, highly abundant or host-associated species.

## Main

The gene content of microbial genomes constantly changes through gain and loss of genes^1^. Gene gain through horizontal gene transfer (HGT) in particular is a driving force in prokaryotic genome evolution^1,2^, and most genes have been shown to undergo HGT at least once in their evolutionary history^3,4^. However, foreign genes can be a burden or even toxic to the recipient^5^, typically persisting only as long as is imposed by fluctuating environmental circumstances. In a simple two-class model of gene evolution^6^, such genes display high rates of turnover. In contrast, other foreign genes may provide sufficient benefit to the recipient, outweighing maintenance costs and persisting long enough to be detected in present-day genomes through computational methods^7^.

Multiple conceptually diverse approaches for computational HGT detection exist^8^. Detecting genomic regions with abnormal sequence composition has the advantage of requiring the recipient genome only. However, such detection is restricted to recent transfer events due to gene amelioration, whereby foreign DNA evolves to resemble that of its host species^9,10^. Alternatively, HGT can be detected through comparing genomes and identifying discrepancies between gene and species evolutionary history. These comparative genomics approaches include the detection of nearly identical sequences in genomes from different species^11–16^ or the more computationally intensive modelling of gene evolution through processes such as gene duplication, transfer or loss^3,4,17–19^. The next-generation sequencing revolution has enabled HGT detection through comparative genomics approaches by enabling an abundance of publicly available, high-quality prokaryotic genomes in curated databases such as proGenomes^20^.

Previous large-scale surveys of HGT across different environments have showcased the contribution of shared ecology to HGT^11,13,14,21^. Generally, inter-environmental transfers were found to be rare, with the possible exception of antibiotic resistance genes^11^. The importance of shared ecology in determining HGT frequency can be explained from two different perspectives. On the one hand, similar environments may exert similar pressures, prioritizing the persistence of specific functional traits. On the other hand, as most HGT mechanisms require physical proximity between the donor and the recipient^22^, co-occurring within the same environment may simply provide more opportunities for HGT.

In this study, we aim to elucidate both ecological and evolutionary factors that contribute to a successful gene gain event through HGT. Using the gene content of 8,790 species’ pangenomes^20^ clustered into over a million gene families, we ran RANGER-DTL to model duplication, transfer and loss events in gene evolution^23^. In parallel, we searched for these species in the MicrobeAtlas database (https://microbeatlas.org/), obtaining more than one million microbial community profiles from diverse, globally distributed environments. By following species presence and abundance profiles across this dataset, we show that co-occurrence, abundance and dispersal patterns all determine HGT success. By looking at functionality and ubiquity of transferred genes, we observe that recent transfers are enriched for genes involved in transcription, replication and repair, and in antimicrobial resistance genes. By comparison, old transfers are enriched for genes involved in amino acid, carbohydrate, and energy metabolism, and are more likely to concern genes that are present in nearly all members of a species. This study provides an overview of global ecological trends in HGT.

## Results and discussion

### Extensive contribution of HGT to prokaryote genome evolution

**Fig. 1:**
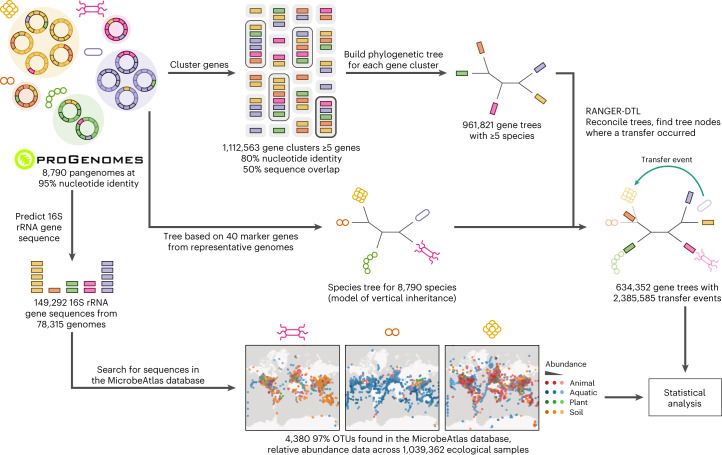
Global-scale computational detection of HGT events and dataset integration with relative abundance profiles from over a million environmental samples. Pangenomes from 8,790 species were generated by clustering coding sequences at 95% nucleotide identity. A toy example depicts the pangenomes of five prokaryotic species: yellow, pink, orange, purple and green. The genomes belonging to these species are depicted as fragmented circles of the corresponding colour, wherein each fragment represents a coding sequence. Coding sequences chosen as representatives in the pangenome are outlined with a darker shade of the same colour. These representative sequences, depicted as colourful rectangles, were then clustered at 80% identity to form gene ‘families’ (semi-transparent grey rectangles, clusters containing at least five genes are outlined). For each cluster containing data from at least five species, a phylogenetic tree was generated and compared with the species tree to detect HGT events. In parallel, 16S rRNA gene sequences were predicted (depicted as rectangles in the colour denoting the species assignment of the corresponding genome) and mapped to OTUs in the MicrobeAtlas database to obtain relative abundance data across ecological samples. Relative abundance data are depicted as points on the MicrobeAtlas sample map, with colours representing the annotated habitat of the corresponding sample (red, animal; blue, aquatic; green, plant; orange, soil) and darker colours reflecting a higher relative abundance of the species of interest. The HGT event data and relative abundance profiles were then integrated for downstream analysis.

**Fig. 2:**
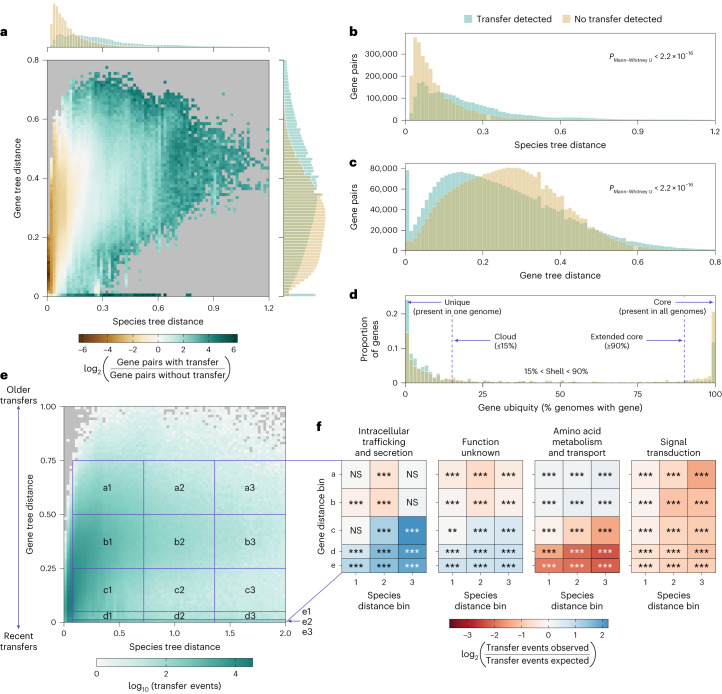
Genes participating in HGT are mostly accessory and display distinct functional profiles depending on time passed since transfer. **a**, Two-dimensional histogram depicting distributions of the distance between genes in a pair (*y* axis, right marginal histogram) against the distance between their corresponding species (*x* axis, top marginal histogram), comparing gene pairs with (green; *n* = 15,561,491) and without (brown; *n* = 3,042,429) transfers. Bins containing fewer than ten observations from each group are coloured in grey. **b**, After normalizing for differences in gene distance distributions, the species distance distribution of gene pairs with transfers (green) is significantly different to that of gene pairs without transfers (brown) (two-sided Mann–Whitney *U-*test, *P* < 2.2 × 10^−16^, *n* = 3,037,896 per group). **c**, After normalizing for differences in species distance distributions, the gene distance distribution of gene pairs with transfers (green) is significantly different to that of gene pairs without transfers (brown) (two-sided Mann–Whitney *U-*test, *P* < 2.2 × 10^−16^, *n* = 2,854,965 per group). **d**, Distribution of gene ubiquity (expressed in the fraction of genomes in species with gene) in putative recipient species for gene pairs with (green; *n* = 335,841) and without (brown; *n* = 40,450) transfers. **e**, Two-dimensional histogram depicting distribution of all transfer events (*n* = 2,385,585) based on average gene distances of all genes involved in the transfer event and average species distances of all corresponding species. **f**, Functional enrichment within bins depicted in **e**. Species distance bins are labelled 1 (0.08–0.72), 2 (0.72–1.36) and 3 (1.36–2.00). Gene distance bins are labelled a (0.50–0.75), b (0.25–0.50), c (0.00–0.25), d (0.00–0.05) and e (0.00–0.01). The significance (postmultiple testing correction) of enrichment (blue) or depletion (red) in the number of transfers is indicated using asterisks within boxes. ***P ≤ 0.001, **P ≤ 0.01, *P ≤ 0.05, two-sided binomial test after multiple testing correction using the Holm–Sidak method. NS, not significant. Source data

**Extended Data Fig. 1:**
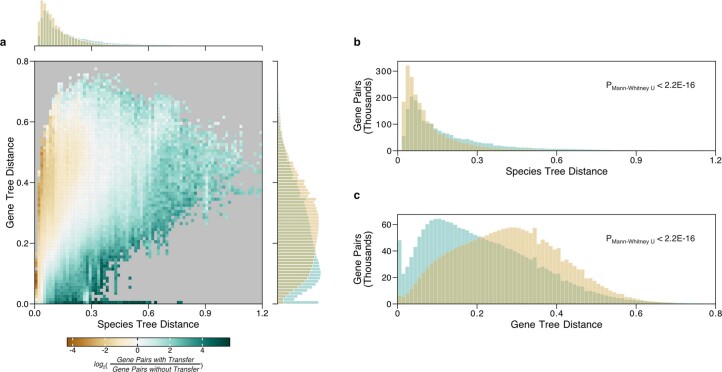
Genes participating in HGT display lower gene distances and originate from more distant species when compared to non-transferred genes. Each transfer event is represented by one randomly-chosen gene pair. (**a**) Two-dimensional histogram depicting distributions of the distance between genes in a pair (y-axis, right marginal histogram) against the distance between their corresponding species (x-axis, top marginal histogram), comparing gene pairs with (green, n = 2,385,585) and without transfers (brown, n = 3,042,429). Bins containing fewer than ten observations from each group are coloured in grey. (**b**) After normalizing for differences in gene distance distributions, the species distance distribution of gene pairs with transfers (green) is significantly different to that of gene pairs without transfers (brown) (two-sided Mann-Whitney U test, *P* ≤ 2.2×10^−16^, n = 2,175,683). (**c**) After normalizing for differences in species distance distributions, the gene distance distribution of gene pairs with transfers (green) is significantly different to that of gene pairs without transfers (brown) (two-sided Mann-Whitney U test, *P* ≤2.2×10^−16^, n = 2,040,229).

To detect HGT events, we first created pangenomes for 8,790 species based on 78,315 high-quality, single-isolate genomes^20^. The resulting 41 million genes were clustered on minimum 80% nucleotide identity and minimum 50% sequence overlap into 22 million clusters, 961,821 (4.4%) of which covered more than 5 species. For each such gene cluster, reconciliation with the species tree based on 40 universal single-copy marker genes^20^ was performed using RANGER-DTL^23^ (Fig. 1 and Methods), resulting in 2.4 million well-supported unique transfer events that involved 8,756 species and 1.7 million species pairs (4.4% of all possible species pairs). Previous studies considering trends in HGT based on thousands of genomes have focused on transfers involving gene pairs with ≥99% nucleotide identity^11,13,15,16^. Such gene pairs comprised 3.1% of detected events in our dataset (see right peripheral histograms in Fig. 2a and Extended Data Fig. 1a for the distribution of HGT events across gene distances). By using tree reconciliation for HGT event detection, we obtained an extended set of gene transfers that allowed us to compare whether transfers that happened earlier in evolution were subjected to the same trends as very recent transfer events. Nevertheless, we observed fewer transferred gene pairs with gene tree distances exceeding 0.6 (Fig. 2a and Extended Data Fig. 1a). Older transfers were more difficult to detect with high confidence and were thus less likely to pass our conservative thresholds for HGT event detection (Methods).

At least one transfer event was detected in 634,352 gene trees out of 961,821 (~66%). The fraction of transferred genes varied between species. For example, a transfer event was detected in 61.5% of the genes considered for *Acinetobacter baumannii*, but only in 19.8% for *Listeria monocytogenes*. Across all species, this resulted in an average of 42.5% (interquartile range, 35.9–50.5%) of genes per species affected by HGT. This number is lower than previously reported estimates of an average of 73% (ref. ^17^) and 81% (ref. ^4^) genes per genome affected by HGT. This discrepancy is probably because we use a stricter threshold to cluster sequences: 80% nucleotide identity as opposed to 30% (ref. ^17^) and 25% (ref. ^4^) amino acid identity. Therefore, we do not capture the oldest transfers considered in these studies^4,17^ but we are able to assess HGT in a much larger dataset and look at more recent transfers.

**Extended Data Fig. 2:**
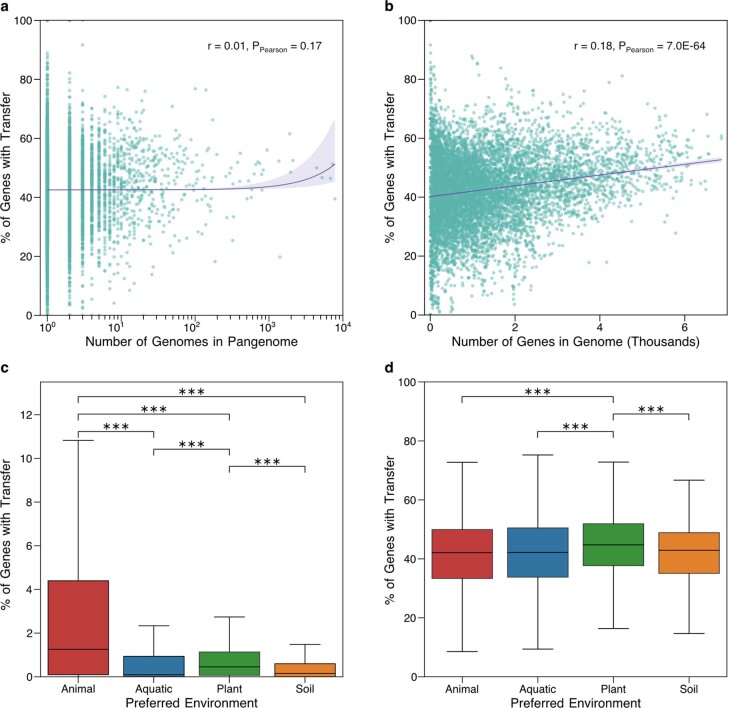
Analysis of factors contributing to interspecies variability in rates of HGT. (**a**) The fraction of genes with a transfer event detected does not significantly correlate with the number of genomes used for generating the pangenome (two-sided Pearson’s product correlation coefficient 0.01, *P* = 0.17, n = 8,776 species). The solid purple line represents the regression line (least squares linear regression in the original space, which results in a non-linear relationship in the displayed log-linear plot). The shaded area represents a 95% confidence interval, estimated based on 1,000 bootstraps. (**b**) The fraction of genes with a transfer event detected correlates with the average number of genes in the genome of the corresponding species (two-sided Pearson’s product correlation coefficient 0.18, *P* = 7×10^–64^, n = 8,776 species). The solid purple line represents the least squares linear regression line. The shaded area represents a 95% confidence interval, estimated based on 1,000 bootstraps. (**c**) The fraction of genes with a recent transfer event (that is involving gene pairs with ≥98% nucleotide identity) detected compared across species’ preferred environments, with *** indicating a significant difference in the fraction of genes with a recent transfer event (two-sided Mann-Whitney U test *P* ≤ 0.001, n = 2,080 animal, 3,047 aquatic, 2,415 plant, 1,153 soil species). (**d**) The fraction of genes with a transfer event detected compared across species’ preferred environments, with *** indicating a significant difference in the fraction of genes with a transfer event (two-sided Mann-Whitney U test *P* ≤ 0.001, n = 2,080 animal, 3,047 aquatic, 2,415 plant, 1,153 soil species). In all depicted boxplots, the line inside the box corresponds to the median. The lower and upper bounds of the box denote the first and the third quartile respectively. Whiskers extend to at most 1.5 times the interquartile range (IQR). Source data

We observed no association (*r* = 0.01, *P*_Pearson_ = 0.17) between the average fraction of transferred genes per species and the number of genomes used for generating the pangenome (Extended Data Fig. 2a). The average fraction of transferred genes was therefore not notably skewed towards better-studied species. Interestingly, the average fraction of transferred genes per species was weakly positively correlated (*r* = 0.18, *P*_Pearson_ = 7.0 × 10^−64^) with the number of genes in the genome (Extended Data Fig. 2b). Previous studies comparing closely related prokaryotic genomes of different sizes have found evidence that HGT is the driving force behind genome expansion, which leads to larger genomes containing a higher fraction of transferred genes^1,2^.

Previous studies have shown host-associated species to exchange more genes than those found in water or soil^11,13,14^, leading us to next investigate the interspecies variability in gene transfer rates from this perspective. We mapped the species in our dataset to operational taxonomic units (OTUs) in the MicrobeAtlas database (Fig. 1), assigning ‘preferred’ habitats based on the highest average relative abundances. Restricting the analysis to transfers concerning gene pairs with ≥98% nucleotide identity, we indeed observed the highest median fraction of transferred genes in animal-associated species (1.32%). Plant-associated species had the second highest median fraction of transferred genes (0.46%), followed by soil-associated (0.16%) and finally water-associated (0.10%) species (Extended Data Fig. 2c). In contrast, when considering all transfer events in the dataset, we found no significant difference between animal-associated, water-associated and soil-associated species (Extended Data Fig. 2d). These findings indicate that on longer evolutionary scales, the loss of transferred genes may compensate for the higher rate of HGT in animal-associated species. Alternatively, animal-associated species may disappear at higher rates, possibly as a result of their host species going extinct.

### Enrichment of accessory genes in recent transfers

We next focused on the distribution of our dataset with respect to species and gene distance (see Fig. 2a for all gene pairs in the dataset and Extended Data Fig. 1a for one representative gene pair per transfer event). The majority of gene pairs in the dataset originated from closely related species (species distance <0.3; Fig. 2a and Extended Data Fig. 1a, top histograms). However, gene pairs with transfer events were more likely to originate from distantly related species when compared with gene pairs without transfer events, especially after subsampling gene pairs with and without transfer events to follow the same gene distance distribution (*P*_Mann–Whitney *U*_ ≤ 2.2 × 10^−16^; Fig. 2b and Extended Data Fig. 1b). In addition, we observed generally lower gene distances in gene pairs with detected transfers, especially after subsampling gene pairs with and without transfer events to follow the same species distance distribution (*P*_Mann–Whitney *U*_ ≤ 2.2 × 10^−16^; Fig. 2c and Extended Data Fig. 1c). These results confirmed that transferred genes are more similar than expected based on species similarity.

**Extended Data Fig. 3:**
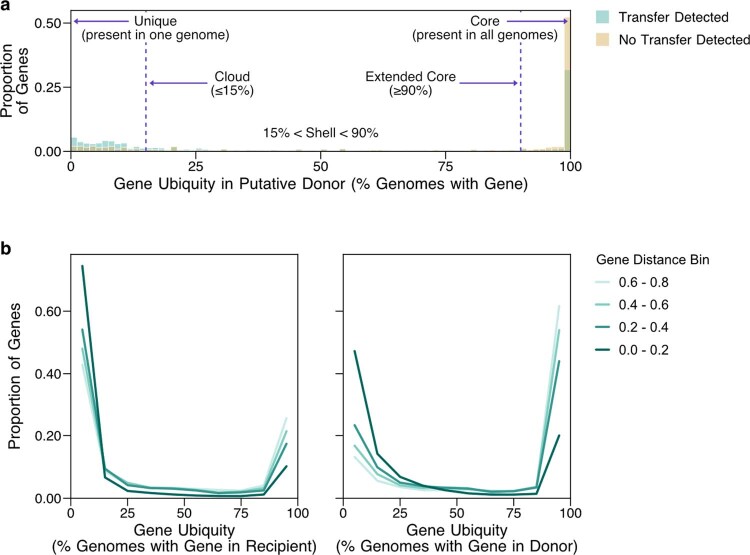
Analysis of gene ubiquity and its relationship to HGT. (**a**) Distribution of gene ubiquity (expressed in the fraction of genomes in species with gene) in putative donor species for gene pairs with (green, n = 335,841) and without (brown, n = 40,450) transfers. (**b**) Comparing gene ubiquity of gene pairs participating in HGT across different gene distance bins. More recent transfer events are depicted in dark green, while old transfers are depicted in light green. The number of gene pairs per bin: 196,527 (0.0–0.2), 76,886 (0.2–0.4), 47,104 (0.4–0.6), 12,324 (0.6–0.8).

The extent of HGT and resulting within-species gene content variation leads to a common distinction between core genes (present in all genomes of a species) and accessory genes (present in some genomes of a species)^24^. Therefore, we studied the ubiquity of genes, namely, how often the transferred genes are found within the pangenome of a species, based on previously defined thresholds for extended core, intermediate-frequency accessory (shell) and low-frequency accessory (cloud) genes^25^ (Fig. 2d). We observed that the odds of encountering a transferred gene within cloud genes were over twice as high as encountering a non-transferred gene within cloud genes (odds ratio = 2.07 in putative recipient species and 2.87 in putative donor species, *P*_Fisher_ ≤ 2.2 × 10^−16^ in both cases; Fig. 2d and Extended Data Fig. 3a). In contrast, the odds of encountering a transferred gene within the extended core genes were over twice as low as encountering a non-transferred gene within the extended core genes (odds ratio = 0.46 in putative recipient species and 0.37 in putative donor species, *P*_Fisher_ ≤ 2.2 × 10^−16^ in both cases). We next used gene distance as a proxy for time since the transfer event because genes transferred earlier in evolution have had more time to accumulate mutations and diverge from the donor. Interestingly, we observed higher fractions of extended core genes in older transfers (Extended Data Fig. 3b), implying persistence of a subset of transferred genes during species evolution. However, core gene sequences may produce more reliable trees than accessory genes (and, indeed, are used for building species trees^26^), increasing the chances of detecting old transfers with high confidence. These results need to be interpreted with caution but they are congruent with the two-class model of gene evolution^6^, whereby genes with high turnover rates can be recruited to perform biological functions with long-term benefit. Such genes then switch to the second, slowly evolving and persistent, class.

### Functional repertoires of recent and old transfer events

**Fig. 3:**
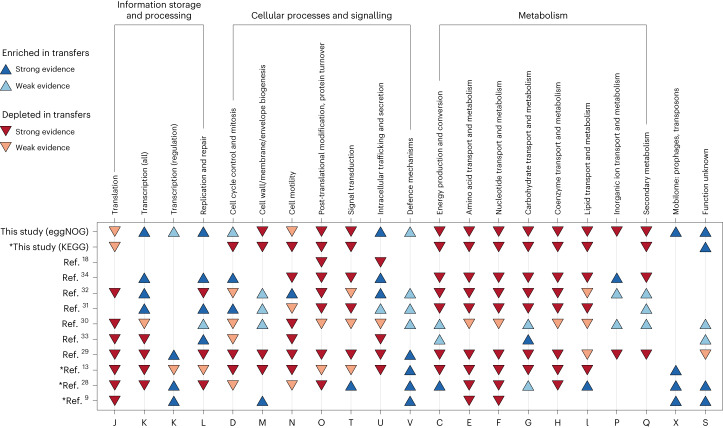
Comparison of functional enrichment analysis results to those from previous studies. The following studies are considered: refs. ^9,13,18,28–34^. Blue upward-facing triangles indicate evidence for enrichment in transfers, whereas red downward-facing triangles indicate evidence for depletion in transfers. Some results from our study (KEGG) and refs. ^9,13,28^ (indicated with an asterisk) do not use COG categories for functional annotation. The letters at the bottom of the figure represent the COG categories, the descriptions of which are given at the top of the figure. For mapping between the different categories used and COG categories, see Supplementary Table 1.

**Extended Data Fig. 4:**
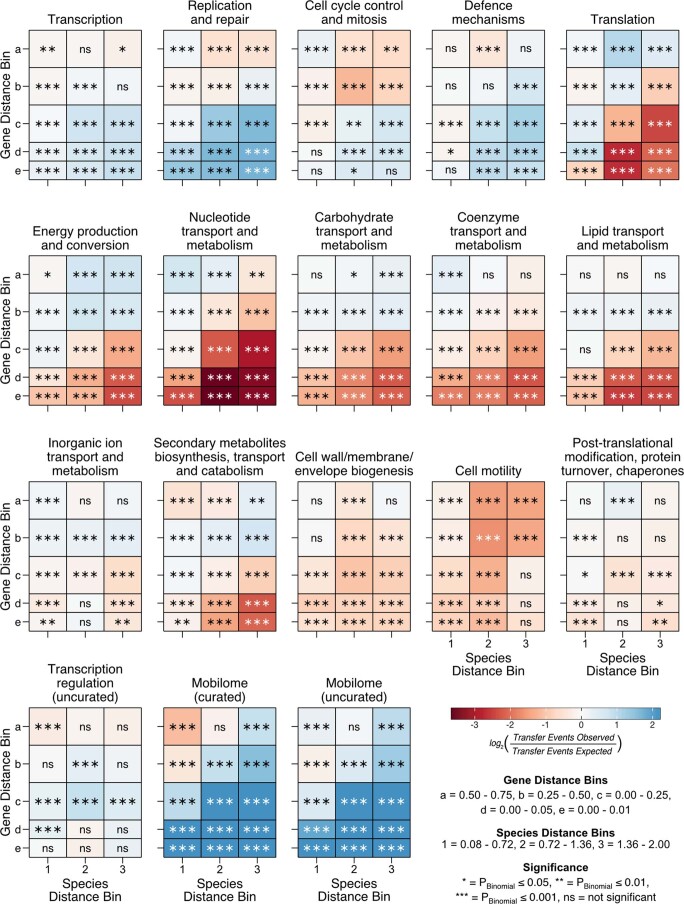
Functional enrichment within bins depicted in Fig. 2e for remaining COG categories not in Fig. 2f. Species distance bins are 1 (0.08–0.72), 2 (0.72–1.36), and 3 (1.36–2.00). Gene distance bins are a (0.50–0.75), b (0.25–0.50), c (0.00–0.25), d (0.00–0.05), and e (0.00–0.01). The significance of enrichment (blue) or depletion (red) in the number of transfers is indicated within boxes (two-sided binomial test after multiple testing correction using the Holm-Sidak method: *** - *P* ≤ 0.001, ** - *P* ≤ 0.01, * - *P* ≤ 0.05, ns - not significant). Source data

**Extended Data Fig. 5:**
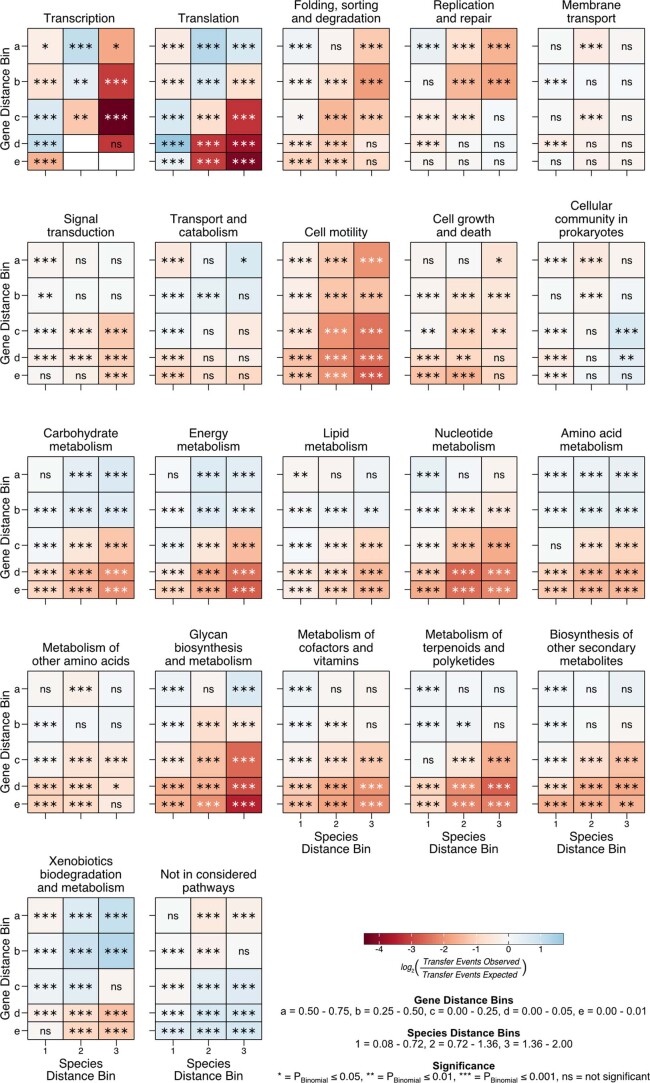
Functional enrichment within bins depicted in Fig. 2e for KEGG pathway maps. Species distance bins are 1 (0.08–0.72), 2 (0.72–1.36), and 3 (1.36–2.00). Gene distance bins are a (0.50–0.75), b (0.25–0.50), c (0.00–0.25), d (0.00–0.05), and e (0.00–0.01). The significance of enrichment (blue) or depletion (red) in the number of transfers is indicated within boxes (two-sided binomial test after multiple testing correction using the Holm-Sidak method: *** - *P* ≤ 0.001, ** - *P* ≤ 0.01, * - *P* ≤ 0.05, ns - not significant). Source data

Multiple studies have considered the function of transferred genes^9,13,15,18,27–34^, which, in the context of very recent transfers, has been shown to be predictive of HGT events^15^. To explore further, we divided our landscape of detected transfer events into bins based on species and gene distance and performed functional enrichment analysis for each bin using the Clusters of Orthologous Genes (COG) categories from eggNOG^35^ (Fig. 2e and Methods), with gene distance again acting as a proxy for time since the transfer event. Recent transfers were enriched for genes participating in defence mechanisms, intracellular trafficking, cell cycle control, transcription, replication and repair, the mobilome, and genes of unknown function (Figs. 2f and 3 and Extended Data Fig. 4). In contrast, genes involved in various metabolic functions were depleted in recent transfer events and enriched in older transfer events (Figs. 2f and 3 and Extended Data Fig. 4). Finally, we found an overall depletion of transfers in genes involved in signal transduction, cell wall biogenesis and cell motility (Figs. 2f and 3 and Extended Data Fig. 4). To validate our findings with another system of functional annotation, we repeated the analysis using KEGG pathways^36^ (Fig. 3 and Extended Data Fig. 5). As most pathways considered were associated with metabolic function, we observed a similar trend of significant depletion in recent transfers and enrichment in older transfers, although the latter was not always statistically significant (Fig. 3 and Extended Data Fig. 5).

The use of various methods for defining transferred genes, different functional annotation systems and choice of background expectation complicate direct comparison between different studies. Moreover, in contrast to most previous studies, we performed functional enrichment analysis separately for gene and species pairs of varying degrees of divergence to prevent recent transfers between closely related species from dominating the enrichment results (Fig. 2e, bottom left). Nevertheless, we were able to select ten previous studies on HGT that performed functional enrichment analysis and compared their results with our observations from recent transfers (gene distance bins c, d, and e; Fig. 3, Supplementary Table 1 and Methods).

Notably, of the broad functional categories analysed for enrichment or depletion in HGT, 12 categories showed over 80% agreement in direction across studies. In cases where there was disagreement between studies (and/or with our study), some of the differences might reflect variations in how the functional categories were defined, or which gene families were particularly amenable to HGT detection. For example, there was low overall consensus in some categories related to information storage and processing, which has been previously discussed to be depleted in HGT^28,30,33^. For such categories, it may be worth looking at the processes with a more fine-grained resolution. For example, in transcription, a case can be made for comparing genes involved in transcription regulation separately from other genes involved in transcription, as these appear to be more consistently enriched in transfers (Fig. 3). Furthermore, as genes can occasionally be transferred together with neighbouring genes on the chromosome, functional classification systems that pay increased attention to operon structures might be particularly suitable in interpreting large-scale HGT trends.

**Extended Data Fig. 6:**
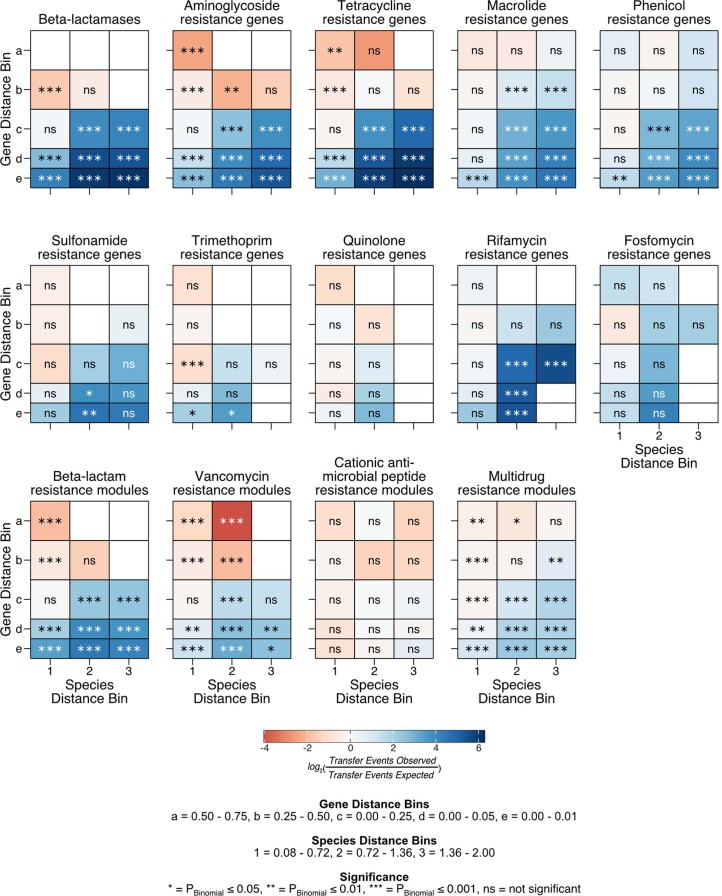
Functional enrichment within bins depicted in Fig. 2e for the BRITE hierarchy ‘Antimicrobial resistance genes’. Species distance bins are 1 (0.08–0.72), 2 (0.72–1.36), and 3 (1.36–2.00). Gene distance bins are a (0.50–0.75), b (0.25–0.50), c (0.00–0.25), d (0.00–0.05), and e (0.00–0.01). The significance of enrichment (blue) or depletion (red) in the number of transfers is indicated within boxes (two-sided binomial test after multiple testing correction using the Holm-Sidak method: *** - *P* ≤ 0.001, ** - *P* ≤ 0.01, * - *P* ≤ 0.05, ns - not significant). Source data

Antimicrobial resistance genes have been previously observed to be transferred at high rates^11,13–16^. Therefore, we focused on genes annotated as such by KEGG. The most recent transfers displayed an over threefold enrichment in genes conferring resistance to β-lactams, aminoglycosides, tetracyclines, macrolides, phenicols and rifamycins (Extended Data Fig. 6). The degree of enrichment increased with species distance, suggesting that aggressive environmental selection for antimicrobial resistance can help overcome mechanistic barriers to HGT^22^ between distantly related species. Apart from the most recent transfers, however, we generally observed a depletion in transfers or no significant signal. The low degree of divergence between antimicrobial resistance genes shared via HGT could indicate transfer event recency but could also stem from strong evolutionary selection acting on these genes. Unfortunately, we are unable to distinguish whether these transfers occurred before or after widespread antibiotic usage, with previous estimates indicating nearly identical genes to have been transferred at any point in the last 1,000 (ref. ^13^) or 10,000 (ref. ^16^) years.

### Co-occurring species are more likely to transfer genes

**Fig. 4:**
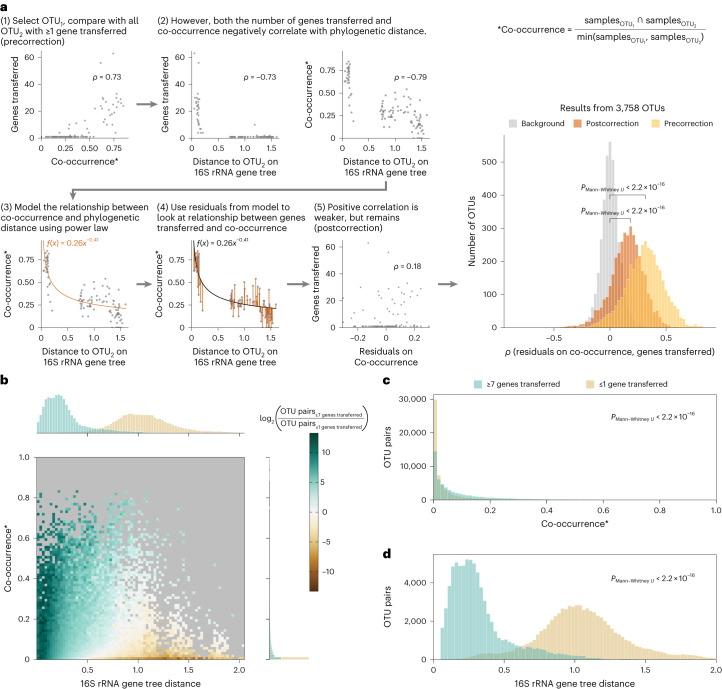
Co-occurring species are more likely to participate in HGT. **a**, Stepwise procedure to correct for the phylogenetic signal contributing to the association between co-occurrence and the number of genes transferred. For each OTU and its partners in HGT, the relationship between co-occurrence and phylogenetic distance is modelled using the power law equation and corrected before correlating the number of genes transferred with co-occurrence. The Spearman correlations (*ρ*) between co-occurrence and the number of genes transferred are significantly greater both precorrection (yellow) and postcorrection (orange) when compared with randomized HGT data (grey) (two-sided Mann–Whitney *U-*test, *P* < 2.2 × 10^−16^, *n* = 3758 OTUs). The formula used for calculating co-occurrence (indicated with an asterisk) is shown on the top right. **b**, Two-dimensional histogram depicting distributions of the co-occurrence between OTUs participating in HGT (*y* axis, right marginal histogram) against their phylogenetic distance (*x* axis, top marginal histogram), comparing OTU pairs with at least 7 genes transferred (green; *n* = 83,725) and OTU pairs with at most 1 gene transferred (brown; *n* = 7,762,564). Bins containing fewer than five observations are coloured in grey. **c**, After normalizing for differences in phylogenetic distance distributions, the co-occurrence distribution of gene pairs with at least 7 genes transferred (green) is significantly different to that of gene pairs with at most 1 gene transferred (brown) (two-sided Mann–Whitney *U*-test, *P* < 2.2 × 10^−16^, *n* = 57,399 per group). **d**, After normalizing for differences in co-occurrence distributions, the phylogenetic distance distribution of gene pairs with at least 7 gene transferred (green) is significantly different to that of gene pairs with at most 1 gene transferred (brown) (two-sided Mann–Whitney *U*-test, *P* < 2.2 × 10^−16^, *n* = 75,620 per group). Source data

We then studied the species participating in HGT and possible associated ecological factors. By using the MicrobeAtlas database, an extensive collection of environmental samples mapped to the same 16S rRNA gene reference collection, we were able to observe the presence of two taxa within the same environmental sample and directly calculate co-occurrence rates. After mapping our dataset to OTUs in the MicrobeAtlas database, we observed a positive correlation between co-occurrence and the number of genes transferred for most OTUs (Fig. 4a, step 1 and precorrection histogram). However, genetic similarity has been shown to influence the success of HGT^11,13,15,16,31^. Indeed, the number of transferred genes negatively correlated with the phylogenetic distance between the OTU pair (Fig. 4a, step 2). In addition, we observed a decrease in co-occurrence with increasing phylogenetic distance, in accordance with closely related taxa preferring similar environments^37^ (Fig. 4a, step 2). We thus sought to correct for the phylogenetic signal in our observations on HGT and co-occurrence.

To this end, we modelled the relationship between co-occurrence and phylogenetic distance using the power law equation (Fig. 4a, step 3). Upon comparing model residuals on co-occurrence with the number of genes transferred, the positive correlation remained for most OTUs (Fig. 4a, steps 4 and 5 and postcorrection histogram). As a complementary approach, we compared species pairs with multiple (seven or more) genes transferred with those with at most one gene transferred with respect to their phylogenetic distance and co-occurrence (Fig. 4b). After normalizing for differences in phylogenetic distance distribution, we observed that pairs of species with multiple transferred genes were significantly more likely to co-occur than pairs of species with at most one transferred gene (*P*_Mann–Whitney *U*_ ≤ 2.2 × 10^−16^; Fig. 4c). Correspondingly, when comparing species pairs with similar degrees of co-occurrence, we observed that pairs with multiple transferred genes were more likely to be closely related (*P*_Mann–Whitney *U*_ ≤ 2.2 × 10^−16^; Fig. 4d).

Observing a positive relationship between co-occurrence and the number of genes transferred, we next asked whether co-occurring species also need to interact to increase the chance of a successful HGT event. Predicting ecological interactions between two species based on co-occurrence can result in spurious associations arising from shared habitats, batch effects or interactions of both considered species with a third intermediary species. To correct for these effects, we used FlashWeave to generate a network of predicted ecological interactions^38^. In brief, FlashWeave uses a Bayesian network-learning approach and interleaved conditional testing to heuristically adjust the associations for potential confounders (Methods). After generating the network, we compared the number of predicted ecological interactions between species pairs with multiple genes transferred and species pairs with at most one gene transferred, subsampling these two groups to follow the same phylogenetic distance and co-occurrence distributions. Within this data subset, we observed 1,012 interactions detected between species pairs with multiple genes transferred and 571 interactions between species pairs with at most one gene transferred, a 1.8-fold enrichment. In contrast, generating a network without conditional testing to remove putative spurious associations yielded 12,525 and 10,071 interactions respectively, a 1.2-fold enrichment. This suggests that there is a notable contribution of ecological interactions to HGT in an environment in addition to mere co-occurrence but further research that considers a larger number of interactions is needed.

### Species with high abundance are more likely to transfer genes

**Extended Data Fig. 7:**
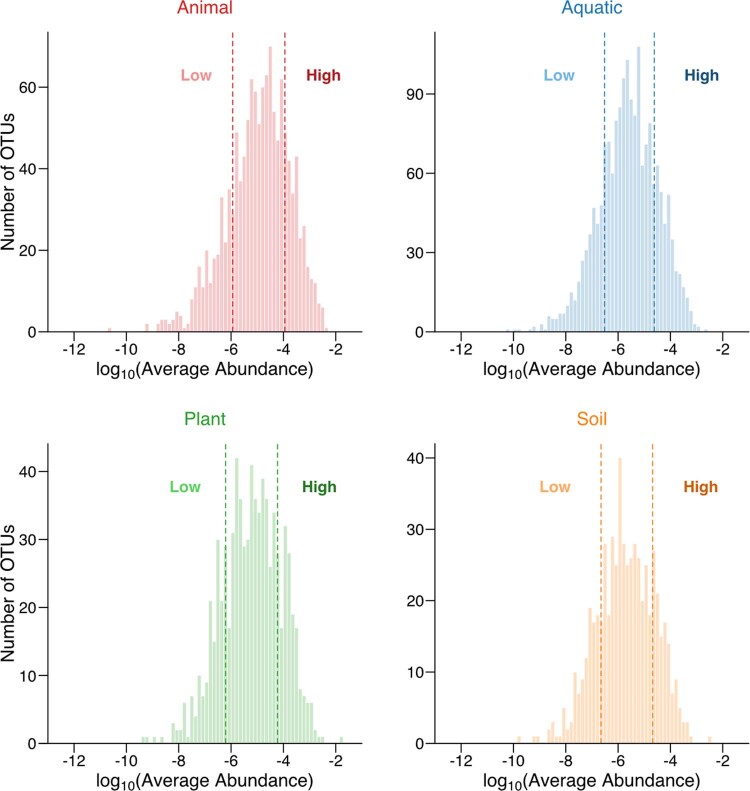
Distribution of OTU abundances in four main environments in the MicrobeAtlas database. Environments considered are: animal (red, n = 1,243 OTUs), aquatic (blue, n = 1,774 OTUs), plant (green, n = 775 OTUs) and soil (orange, n = 588 OTUs). Vertical lines depict the 20% and 80% quantiles of the abundance distribution, with low-abundance OTUs lying to the left and high-abundance OTUs lying to the right. Source data

We used the MicrobeAtlas database not only to look at presence or absence data but also to compare relative abundance profiles across over a million environmental samples. So far, only one study^16^ has looked into the relationship between HGT and species abundance, concluding that abundant bacteria are more likely to transfer genes to other abundant bacteria within the human gut. Unlike this previously mentioned study, we do not possess directly matched cultured isolate genomes with their relative abundance in the corresponding environmental sample, but we can determine whether a species is generally found in high or low abundances within a particular environment. Therefore, we assigned each OTU to its preferred habitat and compared HGT in OTUs lying on opposite ends of the environment’s OTU abundance distribution (Extended Data Fig. 7).

**Fig. 5:**
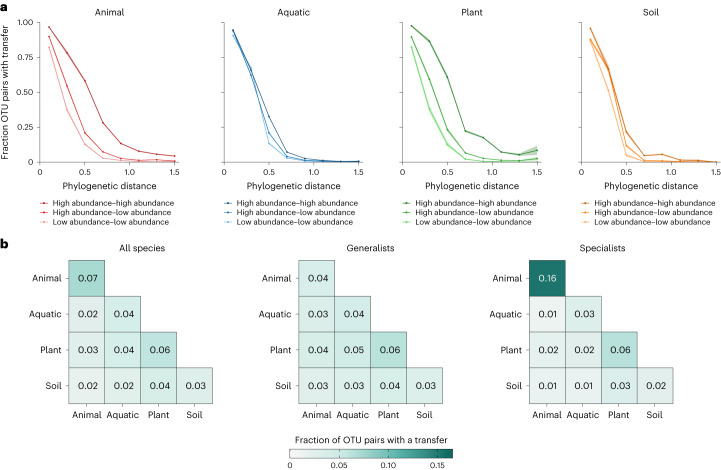
Comparing relative abundance profiles across different environments shows different patterns of HGT for abundant versus rare species and generalists versus specialists. **a**, Comparing the fraction of OTU pairs with a transfer against phylogenetic distance in animal- (shades of red; *n* = 28,385 for high–high OTU pairs, 54,421 for high–low and 27,372 for low–low), water- (shades of blue; *n* = 58,988 for high–high, 91,127 for high–low and 37,504 for low–low), plant- (shades of green; *n* = 11,855 for high–high, 22,082 for high–low and 10,126 for low–low) and soil-associated (shades of orange; *n* = 6,496 for high–high, 12,168 for high–low and 5,553 for low–low) prokaryotes with high (dark shades) and low (light shades) abundance. Error bands are calculated using the Bernoulli principle of uncertainty and depicted in a lighter shade. **b**, Comparison of the fraction of OTU pairs with transfers across the four main environments depicted for all species (left), generalists (centre) and specialists (right). The darker the shade of green, the higher the number of OTU pairs with transfers. Each square contains observations from 9,761 OTU pairs. Source data

We observed a higher fraction of high-abundance OTU pairs participating in HGT when comparing pairs with similar phylogenetic distance (Fig. 5a). Interestingly, the increase in HGT probability with respect to abundance was higher in animal- and plant-associated microorganisms, and was significant in all pairwise comparisons of high–high, high–low and low–low abundance OTUs (Fig. 5a and Extended Data Table 1). In water- and soil-associated microorganisms, the increase in HGT probability was less apparent and not always significant (Fig. 5a and Extended Data Table 1). As HGT mechanisms often require physical proximity between cells exchanging DNA^22^, high-abundance species have more opportunities for transfer, assuming a well-mixed environment. The stronger signal observed in animal- and plant-associated organisms, however, indicates a role for host-associated factors in HGT.

Finally, we defined an index of species generalism based on the relative abundance measurements of OTUs across different environments (Methods). Generalist species can thrive within a wide range of environments, whereas specialist species are confined to a particular environment. Our expectation was therefore that OTUs high on the generalism index can more easily disperse between environments, creating more opportunities for inter-environmental HGT. To this end, we selected 200 OTUs with the highest (generalists) and lowest (specialists) generalism index and compared the number of OTU pairs with at least one transfer event (Fig. 5b). Compared with all species, generalists showed a lower s.d. (*Z*-score, the number of standard deviations from the mean of the corresponding statistic calculated based on all species, = −10.5), lower range (*Z*-score = −6.56) and higher mean (*Z*-score = 2.67) of inter-environmental transfer rates. In contrast, specialists showed a higher s.d. (*Z*-score = 37) and higher range (*Z*-score = 31.4) of inter-environmental transfer rates. Interestingly, we observed a much higher rate of HGT between animal-associated specialists when compared with any other environmental and generalism index combination.

## Conclusion

HGT is extensive and a fundamental driving force in prokaryotic genome evolution. In this study, we performed large-scale computational detection of HGT and integrated these data with an extensive microbial ecology dataset. In our dataset, an average of 42.5% genes in the genome were at one point affected by HGT. Most transferred genes were accessory and probably subjected to high turnover rates. Nevertheless, a fraction of genes transferred earlier in evolution managed to persist and become part of the extended core genome of the species. We have shown that such genes transferred earlier in evolution are enriched for metabolic functions. In contrast, genes transferred most recently are enriched for defence mechanisms and antimicrobial resistance. When considering previous knowledge on HGT and gene function, we show that 9 of 21 COG categories display no consistent signal across studies, suggesting additional factors at play.

Using the MicrobeAtlas database, we followed the global distribution of species that participated in HGT. Even after correction for the confounding effect of phylogenetic relatedness, species co-occurrence rates were positively correlated with larger numbers of transferred genes. In addition, we have shown that species interactions, abundance and dispersal affect HGT rates, indicating the importance of cell proximity for creating opportunities to transfer genes. These ecological factors could not have been assessed on such a global scale with previously available data, showing the value of the MicrobeAtlas database in describing high-level trends in microbial ecology and evolution.

## Methods

### Genome selection and pangenome generation

We based our analysis on the proGenomes v.2.2 dataset containing 82,400 genomes grouped into 11,562 species (that is, specI clusters) that were defined based on 40 single-copy marker genes^20^. The corresponding species tree generated based on concatenated marker gene sequences was kindly provided by the authors of the proGenomes article^20^.

From this initial selection, we filtered out metagenome-assembled genomes, single-amplified genomes, genomes flagged as chimeric by GUNC^39^, genomes that were not taxonomically cohesive with the rest of the specI cluster according to GTDB^26^, genomes with no 16S rRNA gene sequence detected and genomes we could not confidently map to the MicrobeAtlas database (see ‘Mapping genomes to MicrobeAtlas database OTUs’ below). The species tree was pruned to remove these genomes using the ETE Toolkit v.3 (ref. ^40^). As a result, we obtained 78,315 genomes grouped into 8,790 species. For each species, a pangenome was generated by clustering all gene sequences on 95% nucleotide sequence identity as described in ref. ^41^.

### HGT event detection

All gene sequences were clustered using MMseqs2 (ref. ^42^) with minimum overlap of 50%, minimum identity threshold of 80% and clustering mode 0. The rest of the parameters were left as default. For each gene cluster, whenever sequences originated from more than one genome within a species, we only retained sequences that were most similar to those from other species within the gene cluster. We then proceeded with gene clusters containing sequences from at least five different species. Sequences were then aligned using the automatic strategy selection option in MAFFT v.7.471 (ref. ^43^), with all other parameters left as default. On the basis of the multiple sequence alignment, a gene tree was generated using FastTree v.2.1.11 (ref. ^44^) using the generalised time-reversible model^45^ of nucleotide evolution, with all parameters left as default.

Before performing tree reconciliation, we subsampled the species tree using ETE Toolkit v.3 (ref. ^40^) to decrease computational requirements in the following manner: for each gene cluster, the species tree node corresponding to the last common ancestor of all species within the gene cluster was selected. Clades within the species tree not containing any genes from the gene cluster were collapsed for computational efficiency. Subsequently, the subsampled species tree was used to root the gene tree using the OptRoot module from RANGER-DTL v.2.0 (ref. ^23^). We then ran RANGER-DTL with default settings to perform gene and species tree reconciliation for a total of 500×. Gene clusters in which more than 50 optimal roots were detected were not considered further. Reconciliations from each optimal root were aggregated using the AggregateRanger_recipient module from RANGER-DTL v.2.0. We used a custom script to aggregate results across optimal roots and detect tree nodes that were labelled as transfers. For downstream analysis, we considered only transfer events detected in ≥80% reconciliations that contained gene pairs with ≥0.5 minimum branch support in the gene tree. In addition, all multifurcations containing 100% identical genes from different species were considered to be transfer events.

### Calculating the average fraction of genes transferred

For each genome, we counted a gene as having undergone transfer as long as its pangenome-representative gene was involved in a transfer event. For the denominator (that is, total number of genes assessed), we only considered genes if their pangenome-representative genes had passed all steps described above in ‘HGT event detection’. The number of genes transferred was then divided by the total number of genes assessed and the average based on all genomes within a species was calculated. For the examples mentioned in the main text, we used data from specI_v3_Cluster259 for *A. baumannii* and data from specI_v3_Cluster712 for *L. monocytogenes*.

### MicrobeAtlas data retrieval

The NCBI Sequence Read Archive^46^ was searched for samples and studies containing any of the keywords ‘metagenomic’, ‘microb*’, ‘bacteria’ or ‘archaea’ in their metadata. The corresponding raw sequence data (as of 7 March 2020) were downloaded and quality filtered. To assign OTU labels, quality filtered data were mapped to MAPref v.2.2.1 using MAPseq v.1.0 at a ≥0.5 confidence level^47^. We then filtered out samples containing less than 1,000 reads and/or less than 20 OTUs defined at 97% 16S rRNA gene identity and retained samples with at least 90% community coverage (calculated based on the formula in ref. ^48^).

NCBI Sequence Read Archive sample metadata were parsed to classify every sample into four general environments: animal, aquatic, plant and soil. Subsequently, we calculated Bray–Curtis distances between all samples in the dataset and compared community compositions in samples from independent studies. When a sample was consistently similar to samples assigned to a different environment, we adjusted its environment label. In cases where samples with similar community compositions had no general agreement between assigned environments, we removed the environmental label.

### Mapping genomes to MicrobeAtlas database OTUs

We used barrnap^49^ with default settings to predict 16S rRNA gene sequences in the genome selection, proceeding with sequences of ≥50% of expected length. The sequences were then mapped to MAPref v.2.2.1 using MAPseq v.1.0 (ref. ^47^), retaining only sequences that mapped to an OTU with a ≥0.3 confidence level. Genomes containing multiple 16S rRNA gene copies were mapped to OTUs based on a majority rule (≥50% copies) or high confidence (at least one copy with a 0.98 confidence level). Species containing multiple genomes were mapped to OTUs based on majority (≥50% genomes).

### Preferred habitat assignment

For each OTU within the dataset, the average abundance was calculated separately for all samples assigned to the animal, aquatic, plant and soil environments. The OTU was then assigned to its preferred environment based on the highest of the four numbers.

### Gene and species distance normalization

Distances between gene and species pairs were extracted from the corresponding trees using the dist function in ETE Toolkit v.3 (ref. ^40^). To plot the distribution in Fig. 2a, only gene pairs with ≥0.5 minimum branch support values and ≥50% sequence overlap within the multiple sequence alignment were considered. Gene pairs with and without transfer events were normalized with respect to species distance by splitting the species distance distributions into 80 bins and subsampling the group with the larger number of pairs in each bin (either ‘transfer detected’ or ‘no transfer detected’) to the number of pairs in the second group in the corresponding bin (either ‘no transfer detected’ or ‘transfer detected’). The same procedure was performed for normalizing gene pairs with and without transfer events with respect to gene distance. After normalization, the resulting distributions were compared using the two-sided Mann–Whitney *U*-test.

### Pangenome analysis

To calculate gene ubiquity, we counted the number of genomes represented by a gene in each pangenome versus the total number of genomes in the species. For subsequent analysis, only species encompassing ten or more genomes were considered. We used previously defined thresholds^25^ to distinguish extended core genes (≥90% gene ubiquity) and cloud genes (≤15% gene ubiquity). In the species pair participating in HGT, the species with the higher gene ubiquity was labelled as the putative donor, whereas the species with the lower gene ubiquity was labelled as the putative recipient. To compare extended core and cloud genes with or without transfer events, a two-sided Fisher’s exact test was performed.

### Genome annotation and functional enrichment analysis

We used the COG category and KEGG pathway functional annotations provided by the proGenomes database after running eggNOG-mapper for eggNOG 5.0 (ref. ^35^). Each gene cluster was annotated to the corresponding functional categories based on the union of all gene annotations within the cluster. To analyse genes associated with the mobilome, we looked up which terms corresponded to the ‘X—Mobilome: phages, transposons’ category in the database of COGs^50,51^ (mobilome, curated, in Extended Data Fig. 4). In addition, we extracted terms that contained the following keywords in the annotations provided by the proGenomes database: ‘phage’, ‘transposon’, ‘transposase’, ‘transposition’, ‘transposable’, ‘mobile’, ‘mobilization’, ‘integrase’, ‘integration’, ‘plasmid’, ‘conjugative’, ‘conjugation’, ‘transformation’ and ‘competence’ (mobilome, uncurated, in Extended Data Fig. 4). To analyse genes associated with transcription regulation, we extracted terms from the transcription category that contained the following keywords in the annotations provided by the proGenomes database: ‘regulation’ and ‘regulator’ (transcription regulation, uncurated, in Extended Data Fig. 4). We calculated a functional category’s background expectation fraction by counting the total number of genes that passed the pipeline that were annotated to this category divided by the total number of genes that passed the pipeline.

For each detected transfer event, we calculated the average species and gene distance by taking all average pairwise distances between left descendants and right descendants of the transfer event (for gene distance calculations, only gene pairs with ≥50% sequence overlap were considered). The resulting distribution of species and gene distances can be seen in Fig. 2e. For functional enrichment analysis, minimum and maximum species and gene distance cut-offs were selected in such a way that there were no bins without observations, with the resulting area divided into thirds. We also looked specifically at transfer events at the 0.01 and 0.05 gene distance cut-offs (approximately ≥99% and ≥95% sequence identity, respectively) as these results would be more comparable to previous studies that detected HGT events based on nearly identical sequences. We then counted the number of transfer events annotated to each functional category divided by the total number of transfer events in the area. The observed fraction of events annotated to a specific function was then tested with a two-sided binomial test against the fraction of all genes on which the pipeline was run that were annotated to this function. Resulting *P* values were corrected for multiple testing using the Holm–Sidak method.

A similar procedure was performed using KEGG ortholog annotations, grouping them into KEGG pathway maps (09101–09145) for Extended Data Fig. 5 and antimicrobial resistance genes (BR:ko01504) for Extended Data Fig. 6.

### Functional repertoire comparison with previous studies

We compared our functional enrichment analysis results with those from refs. ^9,13,18,28–34^. In most of these studies, functional categories were based on the COG database, with the exception of ref. ^13^ (with categories based on the SEED^52^) and refs. ^9,28^ (both with categories based on TIGRFAMs^53^). The mapping between COG categories and KEGG pathways (used in our study), SEED and TIGRFAMs can be found in Supplementary Table 1.

For our study, we considered enrichment data from the most recent transfers, that is, gene distance bins 0.00–0.01, 0.00–0.05 and 0.00–0.25. These three gene distance bins together with three species distance bins provided us with nine data points to consider for each functional category. We assigned a functional category to have strong evidence for enrichment or depletion in transfers if at least seven of the nine data points showed significant enrichment or depletion. We assigned a functional category to have weak evidence for enrichment or depletion in transfers if most data points showed enrichment or depletion but this was not always statistically significant.

For ref. ^18^, we considered the results depicted in Fig. 8d and Supplementary Table 13 of the article. We calculated the first and third quartiles of the HGT index using all genes in Supplementary Table 13. We assigned a functional category to have strong evidence for enrichment in transfers if the median HGT index from genes in this category was greater than the third quartile. We assigned a functional category to have strong evidence for depletion in transfers if the median HGT index from genes in this category was less than the first quartile. Only functional categories containing at least five genes were considered.

For ref. ^34^, we considered the results depicted in Fig. 9 of the article. We considered only recent HGT events (≥99% nucleotide sequence identity). We assigned a functional category to have strong evidence for enrichment in transfers if the median recent HGTs in this category was greater than the third quartile. We assigned a functional category to have strong evidence for depletion in transfers if the median recent HGTs in this category was less than the first quartile.

For ref. ^32^, we considered the results depicted in Fig. 4a (*HTgenes* row) of the article. We considered a functional category to have strong evidence for enrichment or depletion in transfers if the observed-to-expected ratio of orthologous groups was significantly different from one.

For ref. ^31^, we considered the results depicted in Supplementary Fig. 7 of the article. We considered a functional category to have strong evidence for enrichment or depletion in transfers if the relative proportion of transferred genes was significantly over- or underrepresented when compared with the set of all bacterial genes.

For ref. ^30^, we considered the results depicted in the first two columns of Table 3 of the article. We considered a functional category to be enriched in transfers if its relative transferability was higher than one, and to be depleted in transfers if its relative transferability was lower than one. We used a *P* value cut-off of 0.05 to distinguish strong and weak evidence for enrichment or depletion.

For ref. ^33^, we considered the results depicted in Table 2 of the article. In the table, functional categories were listed that significantly differed from the background of all gene families. We used a *P* value cut-off of 0.05 to distinguish strong and weak evidence for enrichment or depletion.

For ref. ^29^, we considered the results depicted in Fig. 4b of the article. We used *Z*-score cut-offs of 2 and −2 to distinguish strong and weak evidence for enrichment or depletion.

For ref. ^13^, we considered the results depicted in Supplementary File 6 (SEED level 1 and SEED level 2) of the article. We used a *P* value cut-off of 0.05 to distinguish strong and weak evidence for enrichment or depletion. We downweighted depletion evidence for the ‘transcription (regulatory)’ and ‘signal transduction’ categories as they both mapped to ‘regulation and cell signalling’ in the SEED. For COG categories that mapped to multiple categories in the SEED, we indicated evidence based on the consensus from these categories.

For ref. ^28^, we considered the results depicted in Table 2 of the article. We downweighted depletion evidence for ‘cell cycle control and mitosis’ and ‘cell motility’ as they both mapped to the ‘cellular processes’ in TIGRFAMs. We also downweighted enrichment evidence for ‘carbohydrate transport and metabolism’ as there was no one-to-one mapping for this category.

For ref. ^9^, we considered the results depicted in Fig. 2 of the article. We considered a functional category to be enriched in transfers if the proportion of transferred genes was greater than 10%, and to be depleted in transfers if the proportion of transferred genes was less than 3%.

### Co-occurrence analysis

An OTU was detected as present in a given sample if its relative abundance was at least 0.01%. To calculate the co-occurrence between two OTUs, we counted the number of samples in which both OTUs were present and divided it by the number of samples in which the less prevalent OTU was present. Phylogenetic distances between OTUs were retrieved from the MicrobeAtlas database 16S rRNA tree using the dist function in ETE Toolkit v.3 (ref. ^40^).

For modelling the relationship between co-occurrence and phylogenetic distance, we only considered OTUs that exchanged at least 1 gene with 30 other OTUs and OTU pairs in which both OTUs were present in at least 20 environmental samples. The power law equation (1) is as follows:

1 $${{\rm{CO}} \approx k\times {\rm{PD}}^{a},}$$

where CO stands for co-occurrence, PD stands for phylogenetic distance, and *k* and *a* are parameters fitted using the nlstools package in R^54^. Model residuals were then used to calculate Spearman correlations with the number of genes transferred. To generate the background distribution, the number of genes was shuffled before calculating Spearman correlations. The resulting distributions of Spearman correlations generated based on raw co-occurrence (precorrection), model residuals (postcorrection) or background were compared with each other using the two-sided Mann–Whitney *U*-test.

The analysis depicted in Fig. 4b–d has been performed using a similar set-up as described in ‘Gene and species distance normalization’. We used the ≥7 genes transferred cut-off to denote OTU pairs with many transfer events as this corresponded to the 80% quantile of OTU pairs with at least 1 gene transferred.

### Interaction prediction and analysis

Global networks of predicted interactions were computed with FlashWeave v.0.19.0 (ref. ^38^). This method uses the local-to-global learning approach^55^ to learn the skeleton of a Bayesian network encoding putative ecological relationships between species adjusted for ecological or technical confounders. To this end, FlashWeave uses an interleaved testing scheme that (1) heuristically determines likely confounding variables for each pair of species (based on univariate associations and previous iterations of the algorithm), and (2) subsequently tests whether the focal association holds when conditioned on these candidate confounders.

The parameters used for running FlashWeave were as follows: sensitive = false, heterogeneous = true, and max_k = 3 (with confounder correction) or max_k = 0 (without confounder correction). With these settings, FlashWeave converts non-zero read counts to centred log-ratio-transformed values to account for compositionality and discretizes these values. Mutual information tests are then run on the discretized values. We used co-occurrence data from all 95,422 OTUs contained within the environmental sample dataset, filtering the resulting network for edges between the 4,380 OTUs for which transfer event data were generated. OTU pairs with a score higher than zero were considered as interacting. To normalize for differences in phylogenetic distance and co-occurrence distributions between species with at least seven genes transferred and species with zero or one gene transfer, the procedure described in ‘Gene and species distance normalization’ was performed with simultaneous subsampling on phylogenetic distance and co-occurrence for 80 × 80 bins.

### Abundance analysis

We used the same relative abundance numbers as calculated in ‘Preferred habitat assignment’. For each OTU, we only considered its abundance within its preferred environment, denoting high-abundance OTUs as those whose abundance was above the 80% quantile in this environment. In contrast, we denoted low-abundance OTUs as those whose abundance was below the 20% quantile in this environment. OTU pairs were then sorted based on phylogenetic distance and the fraction of OTU pairs with at least one transfer event detected was calculated for each phylogenetic distance bin. Error bands were calculated using Bernoulli’s principle of uncertainty. Resulting fractions were then pairwise compared between the high–high, high–low and low–low groups using a one-sided Wilcoxon rank-sum test. Resulting *P* values were corrected for multiple testing using the Benjamini–Hochberg method.

### Generalist and specialist analysis

We computed a generalism index for each OTU reflecting its environmental flexibility. This index was calculated based on the entropy of the OTU’s abundance values across the four major environments (animal, aquatic, soil and plant). OTUs with similar abundances across environments had higher entropy. OTUs with uneven abundances across environments (a higher abundance in one or a few of the environments compared with the rest) had lower entropy.

To compare inter-environmental transfers, we selected 200 OTUs assigned to each environment (see ‘Preferred habitat assignment’) that displayed the highest entropy (generalists) and 200 OTUs that displayed the lowest entropy (specialists). OTU pairs were then subsampled in such a way that phylogenetic distance distributions were equal between all environments and between generalists, specialists and all species. We then counted the fraction of OTU pairs with at least one transfer event detected. To generate the background expectation, OTU pairs from all species were subsampled to the target phylogenetic distance distribution 1,000×. We then fit a normal distribution to the generated data using the fitdistr function in R^56^ to get an estimate of the expected mean, s.d. and range of transfer rates between different environments.

### Data visualization

Data from Figs. 2 and 4b,c and Extended Data Figs. 1–6 were visualized using seaborn v.0.11.2 (ref. ^57^) and matplotlib v.3.5.1 (ref. ^58^) in Python v.3.7.4. Data from Figs. 3, 4a, and 5 and Extended Data Fig. 7 were visualized using ggplot2 v.3.3.5 (ref. ^59^) in R v.4.1.1.

### Reporting summary

Further information on research design is available in the Nature Portfolio Reporting Summary linked to this article.

### Supplementary information

Supplementary Information Supplementary Table 1.

Reporting Summary

### Source data

Source Data Fig. 2 Statistical source data for Fig. 2f. For Fig. 2a–e, the source dataset is too large to include within the article; please refer to ‘Data availability’ statement.

Source Data Fig. 4 Statistical source data for Fig. 4a. For Fig. 4b–d, the source dataset is too large to include within the article; please refer to ‘Data availability’ statement.

Source Data Fig. 5 Statistical source data. The complete source dataset is too large to include within the article; please refer to ‘Data availability’ statement.

Source Data Extended Data Fig. 2 Statistical source data.

Source Data Extended Data Fig. 4 Statistical source data.

Source Data Extended Data Fig. 5 Statistical source data.

Source Data Extended Data Fig. 6 Statistical source data.

Source Data Extended Data Fig. 7 Statistical source data.

## Figures and Tables

**Extended Data Table 1:**
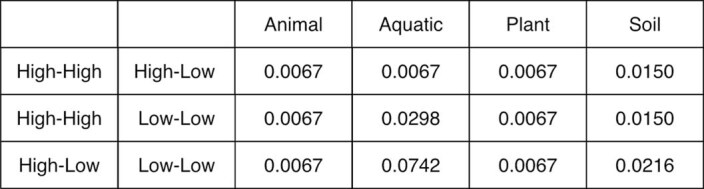
FDR-corrected p-values from one-sided Wilcoxon rank-sum tests on the curves depicted in Fig. 5a

The two curves being compared are indicated in the first two columns.

## Data Availability

The original data from proGenomes v.2 can be downloaded per genome or per specI cluster at http://progenomes2.embl.de. The MicrobeAtlas database is developed within the C.v.M. group and can be downloaded from https://microbeatlas.org/. For the study, we used a subset of an older version of MicrobeAtlas. This subset, along with all datasets generated and used during the study, can be downloaded from 10.6084/m9.figshare.22893632. Source data are provided with this paper.

## Code availability

The scripts used for dataset generation and analysis can be accessed at https://github.com/marydmit/eco_evolutionary_factors_and_hgt.
