# Supplementary material for: A global survey of prokaryotic genomes reveals the eco-evolutionary pressures driving horizontal gene transfer
Source: Nat Ecol Evol. 2024 Mar 5;8(5):986–98. doi: 10.1038/s41559-024-02357-0 (PMC11090817; doi:10.1038/s41559-024-02357-0)
Supplement: Supplementary file 1 — Supplementary Table 1. [file 41559_2024_2357_MOESM1_ESM.pdf]

nature ecology & evolution

Article https://doi.org/10.1038/s41559-024-02357-0

# A global survey of prokaryotic genomes reveals the eco-evolutionary pressures driving horizontal gene transfer

In the format provided by the authors and unedited

**Supplementary Table 1 |** Mapping between COG functional categories and functional categories in KEGG (used in our study), SEED (used in Sheinman M et al), and TIGR (used in Paquola ACM et al and Nakamura Y et al).

| COG Category | KEGG | SEED | TIGR |
|---|---|---|---|
| J: Translation | Translation | Translation (SEED Level 2) | Protein synthesis |
| K: Transcription (all) | Transcription | Transcription (SEED Level 2) | Transcription |
| K: Transcription (regulation) | NA | Regulation and Cell signaling (SEED Level 1) | Regulatory functions |
| L: Replication and repair | Replication and repair | DNA replication; DNA repair (SEED Level 2) | DNA metabolism |
| D: Cell cycle control and mitosis | Cell growth and death | Cell Division and Cell Cycle (SEED Level 1) | Cellular processes: cell division |
| M: Cell wall/membrane/ envelope biogenesis | Glycan biosynthesis and metabolism | Cell Wall and Capsule (SEED Level 1) | Cell envelope |
| N: Cell motility | Cell motility | Motility and Chemotaxis (SEED Level 1) | Cellular processes: chemotaxis and motility |
| O: Post-translational modification, protein turnover and chaperones | Folding, sorting and degradation | Protein processing and modification; Protein degradation; Protein folding (SEED Level 2) | Protein fate |
| T: Signal transduction | Signal transduction | Regulation and Cell signaling (SEED Level 1) | Signal transduction |
| U: Intracellular trafficking and secretion | Membrane transport | Membrane transport (SEED Level 1) | NA |
| V: Defense mechanisms | NA | Virulence, Disease and Defense (SEED Level 1) | Pathogenesis; Toxin production, resistance |
| C: Energy production and conversion | Energy metabolism | Respiration; Photosynthesis (SEED Level 1) | Energy metabolism |
| E: Amino acid transport and metabolism | Amino acid metabolism; Metabolism of other amino acids | Amino Acids and Derivatives (SEED Level 1) | Amino acid biosynthesis |
| F: Nucleotide transport and metabolism | Nucleotide metabolism | Nucleosides and Nucleotides (SEED Level 1) | Purines, pyrimidines, nucleosides, and nucleotides |
| G: Carbohydrate transport and metabolism | Carbohydrate metabolism | Carbohydrates (SEED Level 1) | Central intermediary metabolism |
| H: Coenzyme transport and metabolism | Metabolism of cofactors and vitamins | Cofactors, Vitamins, Prosthetic Groups, Pigments (SEED Level 1) | Biosynthesis of cofactors, prosthetic groups, carriers |
| I: Lipid transport and metabolism | Lipid metabolism | Fatty Acids, Lipids, and Isoprenoids (SEED Level 1) | Fatty acid and phospholipid metabolism |

| | | | |
|---|---|---|---|
| P: Inorganic ion transport and metabolism | NA | Potassium metabolism; Iron acquisition and metabolism (SEED Level 1) | NA |
| Q: Secondary metabolites biosynthesis, transport and catabolism | Metabolism of terpenoids and polvketides; Biosynthesis of other secondary metabolites | Secondary Metabolism (SEED Level 1) | NA |
| X: Mobilome: prophages, transposons | NA | Phages, Prophages, Transposable elements, Plasmids (SEED Level 1) | Plasmid, phage and transposon functions; Mobile and extrachromo-somal element functions |
| S: Function unknown | Not considered in pathways | NA | Unclassified |
