# Supplementary material for: A global survey of prokaryotic genomes reveals the eco-evolutionary pressures driving horizontal gene transfer
Source: Nat Ecol Evol. 2024 Mar 5;8(5):986–98. doi: 10.1038/s41559-024-02357-0 (PMC11090817; doi:10.1038/s41559-024-02357-0)
Supplement: Supplementary file 2 — Reporting Summary [file 41559_2024_2357_MOESM2_ESM.pdf]

nature portfolio

Corresponding author(s): Luis Pedro Coelho, Christian von Mering

Last updated by author(s): Jan 24, 2024

Reporting Summary

Nature Portfolio wishes to improve the reproducibility of the work that we publish. This form provides structure for consistency and transparency in reporting. For further information on Nature Portfolio policies, see our Editorial Policies and the Editorial Policy Checklist.

Statistics

For all statistical analyses, confirm that the following items are present in the figure legend, table legend, main text, or Methods section.

n/a	Confirmed	
☐	☒	The exact sample size (*n*) for each experimental group/condition, given as a discrete number and unit of measurement
☒	☐	A statement on whether measurements were taken from distinct samples or whether the same sample was measured repeatedly
☐	☒	The statistical test(s) used AND whether they are one- or two-sided<br>*Only common tests should be described solely by name; describe more complex techniques in the Methods section.*
☐	☒	A description of all covariates tested
☐	☒	A description of any assumptions or corrections, such as tests of normality and adjustment for multiple comparisons
☐	☒	A full description of the statistical parameters including central tendency (e.g. means) or other basic estimates (e.g. regression coefficient) AND variation (e.g. standard deviation) or associated estimates of uncertainty (e.g. confidence intervals)
☐	☒	For null hypothesis testing, the test statistic (e.g. *F*, *t*, *r*) with confidence intervals, effect sizes, degrees of freedom and *P* value noted<br>*Give P values as exact values whenever suitable.*
☒	☐	For Bayesian analysis, information on the choice of priors and Markov chain Monte Carlo settings
☒	☐	For hierarchical and complex designs, identification of the appropriate level for tests and full reporting of outcomes
☐	☒	Estimates of effect sizes (e.g. Cohen's *d*, Pearson's *r*), indicating how they were calculated

Our web collection on statistics for biologists contains articles on many of the points above.

Software and code

Policy information about availability of computer code

Data collection	Files containing the complete proGenomes v2.2 dataset (Nucleic Acids Res doi: 10.1093/nar/gkz1002, data for corresponding specI clusters and genomes downloadable at: http://progenomes2.embl.de) were kindly provided by the proGenomes v2.2 development team. The MicrobeAtlas database is developed within the Christian von Mering group and downloadable from: https://microbeatlas.org. For the study we used a subset of an older version of the MicrobeAtlas database, which we describe in the Methods section and provide at the link within the data availability statement.
Data analysis	To generate the data set of horizontal gene transfer event data set, the following software was used: MMseqs2, MAFFT v7.471, FastTree v2.1.11, ETE Toolkit v3, RANGER-DTL v2.0 with additional ad hoc python scripts. The pipeline was developed using Nextflow v21.10.0.5640. To map genomes to OTUs within the MicrobeAtlas database, 16S rRNA gene sequences were predicted using barrnap v0.9 and mapped using MAPseq v1.0 to MAPref v2.2.1. To generate interaction networks, FlashWeave v0.19.0 was used.<br><br>For statistical analysis and ad hoc pipeline scripts, Python v3.7 and the following packages were used: Biopython v1.79, glob2 v0.7, h5py v3.2.1, matplotlib v3.5.3, numpy v1.20.3, pandas v1.3.4, scikit-bio v0.5.5, scipy v1.5.3, seaborn v0.12.2, and statsmodels v0.13.2. In addition, R v4.1.1 and the following packages were used: dplyr v1.0.8, fitdistrplus v1.1-8, ggplot2 v3.3.5, gridExtra v2.3, nlstools v2.0-0, plyr v1.8.6, reshape2 v1.4.4, stringr v1.4.0, and tools v4.1.1.<br><br>The code written for the study is accessible at https://github.com/marydmit/eco_evolutionary_factors_and_hgt.

For manuscripts utilizing custom algorithms or software that are central to the research but not yet described in published literature, software must be made available to editors and reviewers. We strongly encourage code deposition in a community repository (e.g. GitHub). See the Nature Portfolio guidelines for submitting code & software for further information.

March 2021

Data

Policy information about availability of data

All manuscripts must include a data availability statement. This statement should provide the following information, where applicable:

- Accession codes, unique identifiers, or web links for publicly available datasets
- A description of any restrictions on data availability
- For clinical datasets or third party data, please ensure that the statement adheres to our policy

The proGenomes data for individual genomes and specI clusters can be downloaded from: http://progenomes2.embl.de.
The current version of the MicrobeAtlas database can be downloaded from: https://microbeatlas.org/index.html?action=download.
A subset of an older version of MicrobeAtlas has been used for the study, which is accessible together with data sets generated and used during the study through the following link: https://doi.org/10.6084/m9.figshare.22893632. Please refer to the README file therein for a comprehensive description of each data set. In case of data derived from the MicrobeAtlas database, the data were subset to only contain information from OTUs covered in the study. The complete data set is available upon request.

Human research participants

Policy information about studies involving human research participants and Sex and Gender in Research.

Reporting on sex and gender	No human research participants were involved in this study.
Population characteristics	No human research participants were involved in this study.
Recruitment	No human research participants were involved in this study.
Ethics oversight	No human research participants were involved in this study.

Note that full information on the approval of the study protocol must also be provided in the manuscript.

Field-specific reporting

Please select the one below that is the best fit for your research. If you are not sure, read the appropriate sections before making your selection.

☒ Life sciences ☐ Behavioural & social sciences ☐ Ecological, evolutionary & environmental sciences

For a reference copy of the document with all sections, see nature.com/documents/nr-reporting-summary-flat.pdf

Life sciences study design

All studies must disclose on these points even when the disclosure is negative.

Sample size	Sample sizes were defined by the availability of data within thresholds used when performing the analyses. The thresholds used for each analysis are described within the Methods section of the manuscript.
Data exclusions	From the proGenomes dataset, we excluded metagenome-assembled genomes, single amplified genomes, chimeric genomes, genomes that were not taxonomically cohesive with the rest of the specI cluster according to GTDB, genomes with no 16S rRNA gene sequence detected, and genomes we couldn't confidently map to an OTU within the MicrobeAtlas database. As a result, 78,315 genomes grouped into 8790 specI clusters were retained.<br><br>From the MicrobeAtlas dataset, we excluded samples containing less than 1000 reads, less than 20 OTUs at 97% 16S rRNA gene identity, and less than 90% community coverage (see https://doi.org/10.1890/11-1952.1 for calculation), retaining 1'039'362 samples as a result.
Replication	No experiments requiring replicate data have been performed as part of the study.
Randomization	To control for covariates, we determined the distribution of a particular covariate within compared groups (for example, that of phylogenetic distance in OTU pairs with many transfers and in OTU pairs without transfers). Observations within compared groups were then randomly subsampled to follow the same distribution with respect to the covariate (or multiple covariates) before testing whether the distributions of the variable of interest were different between compared groups.
Blinding	No analyses conducted in this study required blinding the investigators with respect to group allocation.

Reporting for specific materials, systems and methods

We require information from authors about some types of materials, experimental systems and methods used in many studies. Here, indicate whether each material, system or method listed is relevant to your study. If you are not sure if a list item applies to your research, read the appropriate section before selecting a response.

Materials & experimental systems

n/a	Involved in the study
☒	☐ Antibodies
☒	☐ Eukaryotic cell lines
☒	☐ Palaeontology and archaeology
☒	☐ Animals and other organisms
☒	☐ Clinical data
☒	☐ Dual use research of concern

Methods

n/a	Involved in the study
☒	☐ ChIP-seq
☒	☐ Flow cytometry
☒	☐ MRI-based neuroimaging
